# CM01: a facility for cryo-electron microscopy at the European Synchrotron

**DOI:** 10.1107/S2059798319006880

**Published:** 2019-05-28

**Authors:** Eaazhisai Kandiah, Thierry Giraud, Alejandro de Maria Antolinos, Fabien Dobias, Gregory Effantin, David Flot, Michael Hons, Guy Schoehn, Jean Susini, Olof Svensson, Gordon A. Leonard, Christoph Mueller-Dieckmann

**Affiliations:** a European Synchrotron Radiation Facility, 71 Avenue des Martyrs, 38042 Grenoble, France; b Institut de Biologie Structurale (IBS), Université Grenoble Alpes, CNRS, CEA, 71 Avenue des Martyrs, 38042 Grenoble, France; c European Molecular Biology Laboratory, Grenoble Outstation, 71 Avenue des Martyrs, 38042 Grenoble, France

**Keywords:** cryo-TEM, ESRF, PSB, cryo-EM platform, CM01

## Abstract

Recent improvements in direct electron detectors, microscope technology and software provided the stimulus for a ‘quantum leap’ in the application of cryo-electron microscopy in structural biology, and many national and international centres have since been created in order to exploit this. Here, a new facility for cryo-electron microscopy focused on single-particle reconstruction of biological macromolecules that has been commissioned at the European Synchrotron Radiation Facility (ESRF) is presented. The facility is operated by a consortium of institutes co-located on the European Photon and Neutron Campus and is managed in a similar fashion to a synchrotron X-ray beamline. It has been open to the ESRF structural biology user community since November 2017 and will remain open during the 2019 ESRF–EBS shutdown.

## Abstract 2

CM01 is a recently opened cryo-electron microscopy facility located at the European Synchrotron and is used by the ESRF’s international community for structural biology.

## 1. Introduction   

Recent progress in direct electron detector technology, the provision of intense and coherent electron beams using field emission guns (FEGs and X-FEGs) in cryo-electron microscopes and advances in image-processing algorithms and sample preparation have led to the so-called ‘resolution revolution’ (Kühlbrandt, 2014 ▸; Mitra, 2019 ▸) in cryo-electron microscopy (cryo-EM). Indeed, two thirds of the more than 6000 entries currently in the Electron Microscopy Data Bank (EMDB; Patwardhan, 2017 ▸) have been deposited within the last five years. However, an average resolution for single-particle cryo-EM structures of between 5 and 6 Å for the 3DEM density maps deposited suggests that there is still some way to go before cryo-EM becomes a routine technique for the production of high-resolution structural information. Nevertheless, with this aim in mind, and coupled with significant advances in sample preparation and data processing, many national and international centres for cryo-EM have opened during the last few years (see, for example, Alewijnse *et al.*, 2017 ▸, and references therein; Stuart *et al.*, 2016 ▸; Clare *et al.*, 2017 ▸).

In 2015, the European Synchrotron Radiation Facility (ESRF) took the decision to complement its portfolio of cutting-edge X-ray-based facilities for structural biology (Pernot *et al.*, 2013 ▸; von Stetten *et al.*, 2015 ▸; Linden *et al.*, 2014 ▸; Mueller-Dieckmann *et al.*, 2015 ▸) by acquiring, installing and commissioning a cryo-EM facility (CM01; http://www.esrf.eu/home/UsersAndScience/Experiments/MX/About_our_beamlines/CM01.html) based around a Titan Krios microscope and making it available to its structural biology user community. CM01 is managed as is any other public ESRF beamline, with user access via either peer-reviewed applications or a paid-for proprietary mechanism. CM01 forms part of the Partnership for Structural Biology (PSB; http://www.psb-grenoble.eu) and its operation and development is assured by staff from a consortium of institutes co-located on the European Photon and Neutron Campus (EPN) in Grenoble. Experiments at CM01 are currently restricted to pre-characterized projects for which the sample-freezing conditions have been optimized and initial cryo-EM analyses indicate a high potential for obtaining information to the secondary-structure level. In the intermediate future it is planned that this restriction will be removed and that access to CM01 will be based on scientific merit alone. Here, preliminary sample screening will be carried out using the PSB cryo-EM platform (http://www.ibs.fr/research/research-groups/methods-and-electron-microscopy-group/electron-microscopy-platform/) microscopes located at the Grenoble Institut de Biologie Structurale (IBS). Once this mode of operation is fully functional, it will provide a unique opportunity for scientists of the ESRF’s international community with limited or no access to EM facilities to access the technique for scientifically important projects.

## 2. General setup and infrastructure   

**Figure 1:**
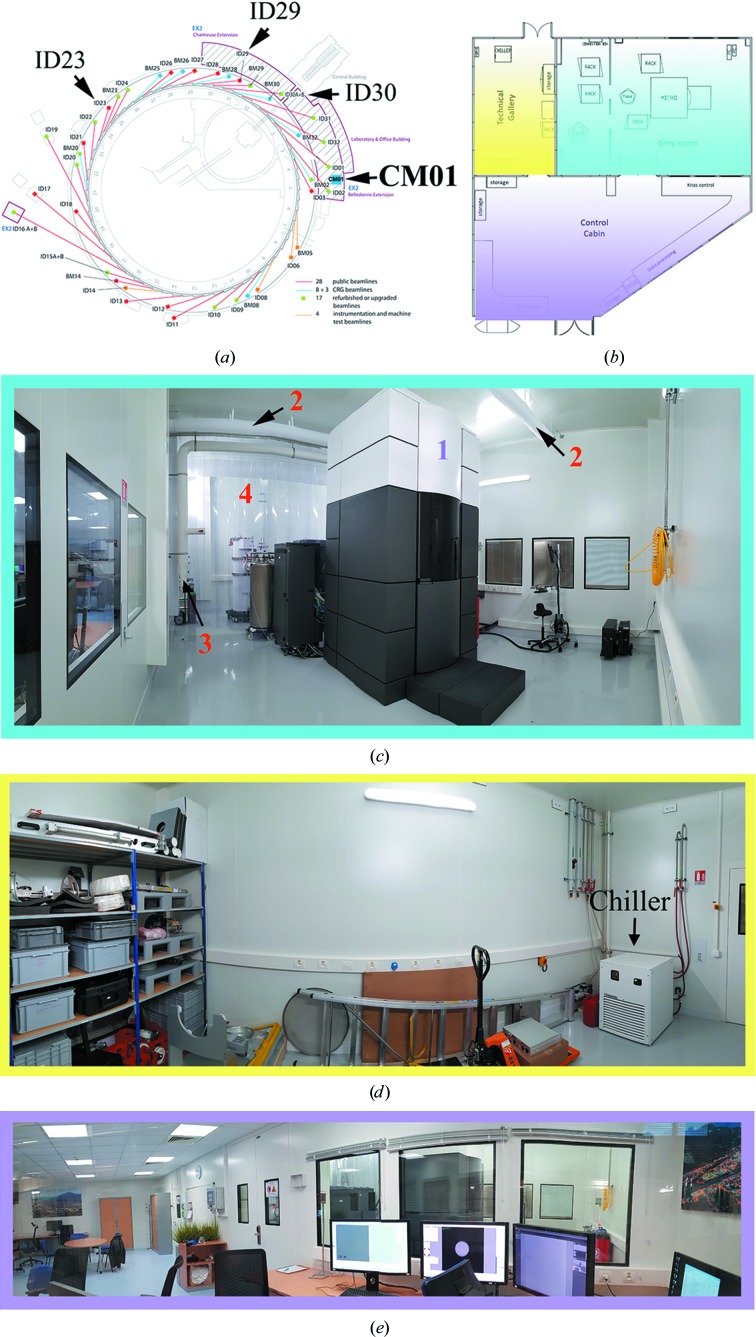
CM01 infrastructure. (*a*) Location of CM01 in the Belledonne extension of the experimental hall of the ESRF. (*b*) Schematic top view of CM01 showing the experimental hutch (EH) in blue, the technical gallery in yellow and the control cabin (CC) in violet. (*c*) The CM01 experimental hutch (grey room) showing the Titan Krios microscope (1), the duct sock porous mesh for uniform air flow (2), SF6 extraction (3) and the acoustic damping sheets (4) behind which the high-voltage generator is located. (*d*) Technical gallery, where the chiller unit (indicated) is placed. (*e*) The large CM01 control cabin.

CM01 is sited on the ‘golden slab’ of the Belledonne extension of the ESRF experimental hall (Fig. 1 ▸ *a*), which was constructed as part of Phase 1 of the ESRF Upgrade. The location was chosen following preliminary measurements confirming that it would provide a stable, vibration-free environment coupled with minimal variations in magnetic field (Table 1 ▸) that might otherwise be associated with installing a cryo-electron microscope in the experimental hall of a synchrotron source. The infrastructure (Figs. 1 ▸ *b*–1 ▸ *e*) in which CM01 is hosted comprises a grey room (ISO8 level) air-conditioned with a temporal thermal stability of 20 ± 0.1°C within 30 min and ±0.8°C per 24 h, a technical cabinet and an experimental control cabin. This room also contains the high-voltage generator, electronics and fluidics cabinets as well as the computer and processor units for the detector. These are screened from the microscope itself (Fig. 1 ▸ *c*). All walls and the ceiling are acoustically damped to avoid any external disturbances, while the water-chilling unit that is needed to cool the electro-magnetic constant power lenses of the microscope is located in a technical cabinet (Fig. 1 ▸ *d*) outside the experimental hutch, again for acoustic and vibrational insulation reasons.

The microscope around which CM01 is built is a Titan Krios G3 (ThermoFisher Scientific; https://www.fei.com) currently operated at its maximal voltage of 300 kV and equipped with a GIF Quantum LS energy filter coupled to a Gatan K2 summit direct electron-counting camera. The microscope also features a Volta phase plate (VPP), which increases the contrast and as such enables the high-resolution imaging of smaller macromolecules of less than 150 kDa (Danev & Baumeister, 2016 ▸).

CM01’s spacious control cabin is equipped with the computing infrastructure required to control both the microscope and the detector (Fig. 1 ▸ *e*) and also includes infrastructure for data processing and backup. Before each experiment, the microscope is set up and aligned to reduce the astigmatism and coma values to less than 0.5 µm (*AutoCTF*) by the facility personnel, who also clip the grids if necessary, load them into the cassette and then into the microscope. The microscope is operated in EFTEM mode with a nanoprobe for data acquisition. Currently, automatic data collection for single-particle reconstruction is carried out using the manufacturer’s control software package *EPU* v.1.11 (ThermoFisher). A typical user session involves 4 h for grid screening (up to six grids) followed by the selection of the best grid squares and hole positions for image acquisition. Many experiments are carried out at a nominal magnification of 130 000 (pixel size of 1.056 Å) using a detector flux density of 5 e^−^ per pixel per second (usually denoted the ‘dose rate’ in EM) and exposure times of 8 s per movie (40 frames per movie), resulting in an accumulated fluence of 40 e^−^ Å^−2^ (usually denoted as the ‘dose’ in EM). Although the setting up and launching of image acquisition is carried out by the CM01 staff, the data-collection strategies are discussed directly with the users, who are either present on-site or access the facility remotely (see below). Using the current setup, the throughput is about 75–80 movies per hour for collection in counting mode for grids with a 2 µm hole size (five movies per hole).

## 3. Computing infrastructure, data pre-processing and presentation of data using EXI/ISPyB   

**Figure 2:**
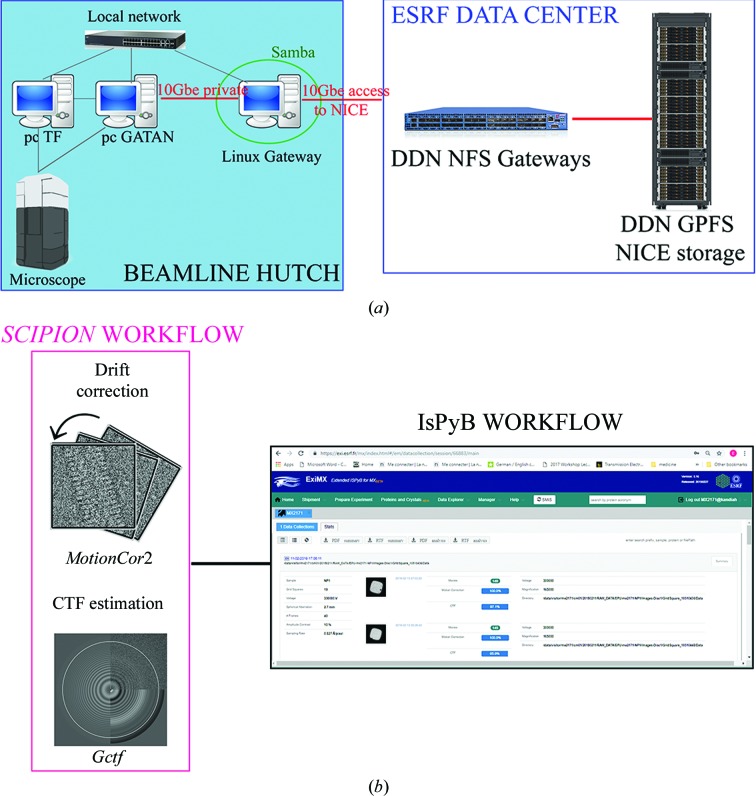
(*a*) Schematic of the IT infrastructure at CM01 for data collection and transfer connections between computers (pc TF, pc GATAN and the microscope are connected via ethernet). (*b*) The single-particle cryo-EM image pre-processing workflow currently carried out at CM01. The collected movies are motion-corrected and CTF values are estimated. The obtained metadata are then accessed and uploaded to the EXI web interface.

The cryo-EM facility greatly benefits from the computing and network infrastructure available at the ESRF for experiments on its X-ray beamlines (Fig. 2 ▸ *a*). Images or movies recorded by the detector are directly transferred to the ESRF central storage system (NICE, Networked Interactive Computing Environment; https://www.esrf.eu/Infrastructure/Computing/NICE) using a dedicated fibre-optic connection (10 Gb s^−1^). This facilitates not only fast data transfer but also allows both on-the-fly data backup onto an external hard drive and image pre-processing. In general, raw data are maintained on disk for up to 50 days from the experiment date and are then archived on tape and stored without time limit. Data backup to external hard disk drives is usually carried out by the users using the same easy-to-use interface that is available on all ESRF beamlines for structural biology. Alternatively, users can also download and transfer data to their home storage system via an rsync command. CM01 implements the ESRF data policy (http://www.esrf.fr/datapolicy).

**Figure 3:**
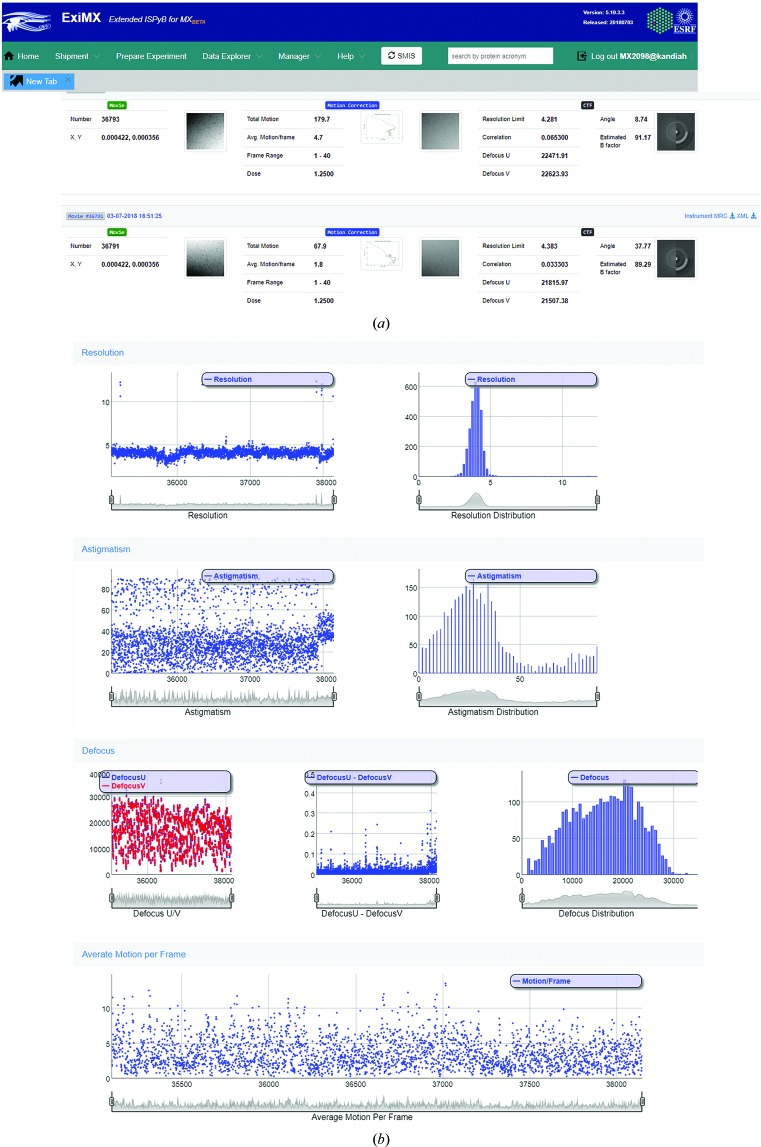
The EXI web interface (https://exi.esrf.fr) provides a summary of the data-collection parameters and pre-processing results on the fly. (*a*) Snapshot of the pre-processing results showing the unaligned average (first column), motion-corrected average (second column) and CTF estimation (third column) images and associated values. (*b*) Overview of data quality. The first three rows show the image series plots and histograms for the information limit in the micrographs, the astigmatism angle and defocus *U* and *V* (astigmatism in the middle frame), respectively. The last plot indicates the average motion per frame in micrometres from the alignment of the acquired movies.

Parallelized image pre-processing (Fig. 2 ▸ *b*) using several GPU-equipped computing clusters is automatically launched on the collected movies using the *SCIPION* wrapper (Conesa Mingo *et al.*, 2018 ▸; Gómez-Blanco *et al.*, 2018 ▸). The imported movies are drift-corrected using *MotionCor*2 (Zheng *et al.*, 2017 ▸) and contrast-transfer function (CTF) parameters are estimated using *Gctf* (Zhang, 2016 ▸). With the current GPU infrastructure, which is subject to constant upgrades, and for an incoming rate of up to 80 movies per hour, pre-processing results are calculated with almost no delay. The output of *MotionCor*2 and *Gctf* are uploaded into the ISPyB database (Delagenière *et al.*, 2011 ▸) and are displayed, along with other metadata, as snapshots in EXI (Fig. 3 ▸; https://exi.esrf.fr). Several relevant parameters, including maximum resolution and astigmatism, are constantly uploaded and plotted both in EXI and on a separate screen inside the control cabin, allowing on-the-fly quality control of incoming data. In the imminent future, extension of the existing data-processing pipeline to include automated or semi-automated particle picking and the generation of 2D classes are foreseen.

## 4. Sample-preparation laboratory and storage facility   

**Figure 4:**
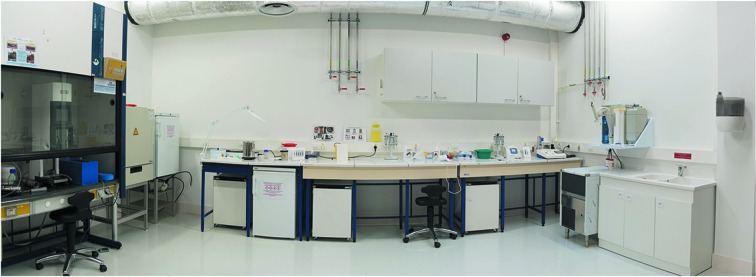
Laboratory access is provided to users for cryo-EM sample preparation. The laboratory includes a Vitrobot positioned in the fume hood, as can be seen on the left side.

As noted above, experiments at CM01 are currently restricted to pre-characterized samples for which frozen grids have been prepared. However, and in preparation for the operation phase of CM01, in which preliminary sample screening and sample optimization will be carried out using the PSB cryo-EM platform, a fully equipped sample-preparation laboratory is associated with the facility (Fig. 4 ▸). The equipment in this laboratory comprises a ThermoFisher Scientific Mark IV Vitrobot and all of the tools necessary for grid preparation, including a glow-discharge apparatus (Ted Pella easyGlow). Users currently applying for microscope time to analyse grids based on pre-characterization can ask for access to this laboratory during the application procedure (see below).

External users of CM01 have the option of either coming to the ESRF to oversee their experiments or sending samples for remote and mail-in data collection. In both cases, cryo-EM grids can be sent to the ESRF prior to the experiment date and stored using individually barcoded grid-boxes provided by the ESRF (https://www.mitegen.com/product/mitegen-cryo-em-pucks-generation-2-0/) in a dedicated storage area. As for experiments on the ESRF’s X-ray-based structural biology beamlines, samples must be registered using the ISPyB sample-tracking module (Delagenière *et al.*, 2011 ▸). In the case that more data need to be collected from a set of grids, samples can be stored for up to six months, a period that can be prolonged upon request. Again, as for the ESRF’s X-ray-based structural biology beamlines, if users do not come to the ESRF for their experiment then the costs of sample transport to and from Grenoble are met by the ESRF (see https://www.esrf.eu/UsersAndScience/Experiments/MX/How_to_use_our_beamlines/Prepare_Your_Experiment/Dewar_sending).

## 5. How to apply for access   

Currently, CM01 delivers ∼600 8 h shifts per year for external user experiments. Experiments are scheduled on a three-day, two-day or one-day basis according to the beamtime demanded in the proposal, with the vast majority of experiments using three days. 10% of the beamtime given is dedicated to in-house research. One day per week is reserved for microscope maintenance and the remaining time is used for commissioning (including service interventions and upgrades) and developments on the microscope. As for all of the ESRF’s facilities for structural biology, access to CM01 can be on either a public or a proprietary basis. For the former, applications, peer-reviewed by the ESRF’s MX Beam-time Allocation Panel (MX-BTAP) comprising several cryo-EM specialists, can currently be submitted at any time via the ESRF’s Rolling Access Mechanism (http://www.esrf.eu/UsersAndScience/UserGuide/Applying/MXApplications). For accepted proposals, the ESRF provides financial support for either user travel to Grenoble (including accommodation and subsistence) or for sample transport to/from Grenoble. Aside from this core mission of providing user service, hands-on workshops on sample preparation (see, for example, http://www.esrf.eu/cryo-em2019-1-workshop.html) as well as on theoretical principles of cryo-EM are held on a regular basis. The normal waiting time from proposal submission until experiment is currently around two months. Proprietary access to CM01 is via the ESRF’s Business Development Office (BDO; http://www.esrf.fr/Industry/contact-industrial-services).

## 6. Microscope benchmarking   

**Figure 5:**
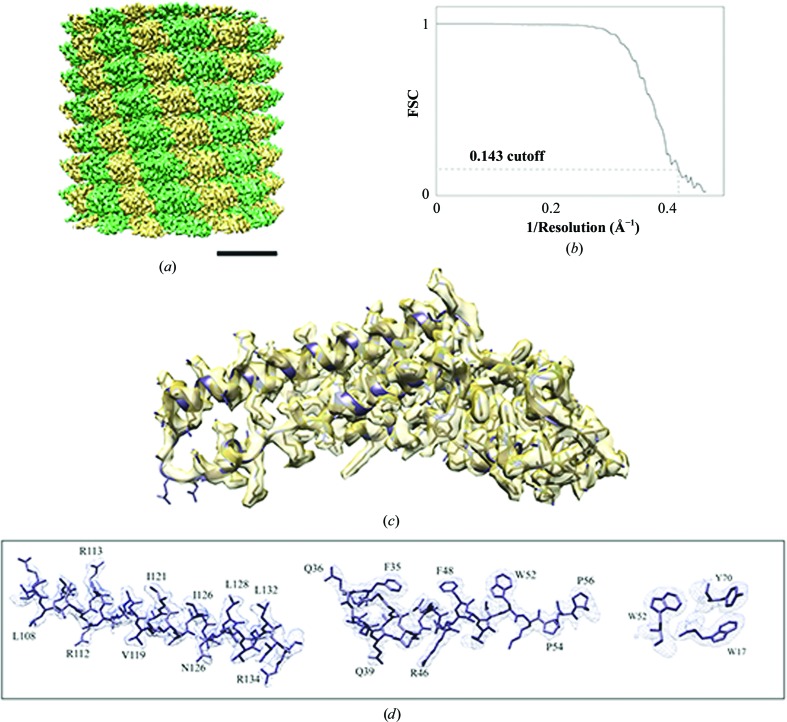
3D reconstruction of TMV particles. (*a*) 3D reconstruction of TMV. Alternate subunits are coloured in yellow and green. The scale bar represents 5 nm. (*b*) Masked-corrected Fourier shell correlation (FSC) curve for resolution. (*c*) An asymmetric unit modelled in the electron density. (*d*) Some examples representing the quality of the EM density and the model.

In order to benchmark the microscope installed on CM01, we used Tobacco mosaic virus (TMV) as a test sample for single-particle reconstruction. TMV grids were prepared using the Vitrobot by pipetting 3.5 µl of 3 mg ml^−1^ TMV onto Quantifoil 1.2/1.3 400 mesh grids (https://www.quantifoil.com/); excess liquid was blotted off for 2 s. The K2 detector was used in counting mode for automatic data collection using the *EPU* software. Movies were collected as gain-normalized and unpacked MRC files at a magnification of 130 000, yielding a calibrated pixel size of 1.067 Å. Drift correction was carried out using *MotionCor*2 (Zheng *et al.*, 2017 ▸) and CTF estimation was performed by *Gctf* (Zhang, 2016 ▸). Further image-processing steps were performed using *RELION* 2.1 (Kimanius *et al.*, 2016 ▸; Scheres, 2012 ▸). Segments were automatically picked and extracted with about 90% overlap between segments. 2D reference-free classification was performed and class averages showing high-resolution features were selected and included for 3D refinement. As a final step, local refinement of the CTF parameters was performed using *Gctf* (Zhang, 2016 ▸). The map was further post-processed by automatically applying a *B* factor of −83 Å^2^, yielding a gold-standard FSC (Henderson *et al.*, 2012 ▸) resolution of 2.3 Å (Fig. 5 ▸), one of the highest resolutions for the TMV structure to be published (Weis *et al.*, 2019 ▸). Even though the resolution of the TMV structure presented here is not the highest to be reported (Song *et al.*, 2019 ▸), the result obtained clearly shows the very high quality of data that can be obtained using CM01. The final helical parameters are 1.408 Å for the helical rise and 22.03° for the helical twist. One TMV protomer (PDB entry 4udv; Fromm *et al.*, 2015 ▸) was rigid-body fitted into the density map using *UCSF Chimera* (Pettersen *et al.*, 2004 ▸) and the atomic positions were further refined using *PHENIX* real-space refinement (Afonine *et al.*, 2018 ▸). The final model fits well into the density with very good statistics (Table 2 ▸). The TMV protomer modelled consists of 153 residues (residues 65 and 98–102 were not visible). Most of the side-chain densities are well defined (Fig. 5 ▸) and only 17% of residues exhibit poorly defined side-chain density. Of these, about 40% are negatively charged amino acids such as aspartic and glutamic acid residues, which are prone to radiation damage (Hattne *et al.*, 2018 ▸).

## 7. The first year of operation   

**Figure 6:**
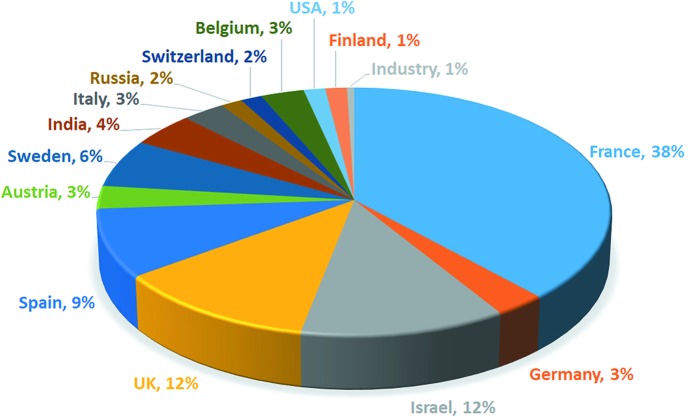
Breakdown of the shifts delivered in 2018 by proposal country of origin for experiments at CM01.

The vast majority of users are from academic institutions located in the 13 ESRF member states (Fig. 6 ▸). At the time of writing, seven publications based, in whole or in part, on data collected at CM01 have already been published. These include different states of the 5HT3 receptor, which allow a description of its activation cycle upon binding to its serotonin substrate (Polovinkin *et al.*, 2018 ▸), the structure of the membrane complex (MC) of a type VI secretion system (T6SS), providing insights into the mechanism of action of the MC complex during T6SS assembly (Cherrak *et al.*, 2018 ▸), the natural tetrameric structure of human butyrylcholinesterase (BChE; Boyko *et al.*, 2019 ▸), the structure of Hantaan virus (Arragain *et al.*, 2019 ▸), the structure of the human ferritin–transferrin receptor 1 complex (Montemiglio *et al.*, 2019 ▸) and the structure of adenovirus type 3 fibre with its receptor desmoglein 2 (Vassal-Stermann *et al.*, 2019 ▸). However, given that most of the experiments carried out are rather challenging, no reliable statistics on publications per year can yet be given.

## 8. Perspectives   

With the rapid growth of the cryo-EM community in the last few years, the demand for data collection at high-end microscopes such as the Titan Krios installed at CM01 has skyrocketed. Since its installation, CM01 has proved to be a highly successful facility, acting in concert with the ESRF’s X-ray-based beamlines for structural biology as a source of primary or complementary structural information in a wide range of projects. One of the major driving forces for the creation of CM01 was to provide a facility that can be accessed by all of the ESRF’s international structural biology user community. However, a lack of infrastructure for sample preparation and screening remains a bottleneck, particularly for researchers in smaller laboratories. To address this and to broaden the accessibility of cryo-EM to researchers working in ESRF member states, the PSB Cryo-EM platform will, for projects approved by the ESRF MX-BTAP, soon provide additional services to users based on one of the following models: (i) the screening of frozen grids and a preliminary data collection if negative-stain characterization has already been carried out and (ii) sample characterization using negative-staining methods followed by cryo-EM sample preparation and screening. If the above steps are successful, data collection using the Titan Krios at CM01 will follow. The ESRF will also continue its training workshops for external users on both theoretical principles and the practical aspects of sample preparation.

## Figures and Tables

**Table 1:** Vibrational and electromagnetic measurements inside the experimental hutch of CM01 The mechanical vibrations are measured in units of *g* (the acceleration owing to gravity in m s^−2^) in three directions approximately at the position of the column at the floor level. Both the AC and DC electromagnetic fields (EMF) are measured in nanotesla p/p in three directions to the column position for three different heights [the GIF approximately 0.5 m from the floor, the compustage approximately 1.5 m from the floor and the gun (source) approximately 2.5 m from the floor].

	Front to back	Left to right	Vertical
Mechanical vibration	1.1 (1.56 µm s^−1^)	1.1 (1.56 µm s^−1^)	2.3 (3.2 µm s^−1^)
EMF AC			
GIF level	42 (50 nT)	21 (50 nT)	29 (75 nT)
Stage level	43 (80 nT)	20 (80 nT)	33 (80 nT)
Source level	42 (80 nT)	21 (80 nT)	39 (80 nT)
Filtered			
GIF level	19 (50 nT)	18 (20 nT)	23 (75 nT)
Stage level	—	—	—
Source level	—	—	—
Near DC			
GIF level	14 (50 nT)	7 (50 nT)	19 (75 nT)
Stage level	6 (80 nT)	11 (80 nT)	19 (80 nT)
Source level	3 (80 nT)	12 (80 nT)	58 (80 nT)

**Table 2:** Data-collection and refinement statistics for the single-particle reconstruction of TMV

Data collection	
Accelerating voltage (kV)	300
Nominal magnification	130 000
Calibrated pixel size (Å)	1.067
Total No. of movies collected	357
Total fluence (e^−^ Å^−2^)	40
No. of frames	40
Defocus range (µm)	−1.0 to −3.0
Data processing	
Final segments	109763
Helical parameters	
Twist (°)	22.04
Rise (Å)	1.41
Gold-standard resolution (Å)	2.3
*MolProbity* statistics	
All-atom clashscore	2.41
Ramachandran plot	
Outliers (%)	0
Allowed (%)	3.97
Favoured (%)	96.03
Rotamer outliers (%)	0
C^β^ deviations (%)	0
Deviations from ideal values	
Bond lengths (µm)	0.007
Bond angles (°)	1.030
Chirality (Å^3^)	0.060
Planarity (°)	0.009
Dihedral (°)	8.534
Map–model CC	
Main chain (760 atoms)	0.68
Side chain (513 atoms)	0.63
EMDB ID	EMD-4928
